# Explainable machine learning model for predicting the outcome of acute ischemic stroke after intravenous thrombolysis

**DOI:** 10.3389/fneur.2025.1668816

**Published:** 2025-09-26

**Authors:** Fanhai Bu, Runlu Cai, Wei Zhang, Xiaohong Tang, Guiyun Cui, Xinxin Yang

**Affiliations:** ^1^Department of Neurology, The First Clinical College, Xuzhou Medical University, Xuzhou, China; ^2^Department of Neurology, Affiliated Hospital of Xuzhou Medical University, Xuzhou, China; ^3^Department of Anesthesiology, The First Affiliated Hospital of Xi'an Jiaotong University, Xi'an, China; ^4^Department of Neurology, Hongze District People's Hospital, Huaian, China

**Keywords:** acute ischemic stroke, intravenous thrombolysis, machine learning, outcome, SHapley Additive exPlanations

## Abstract

**Introduction:**

Acute ischemic stroke (AIS) patients often experience poor functional outcomes post-intravenous thrombolysis (IVT). Novel computational methods leveraging machine learning (ML) architectures increasingly support medical decision-making. We aimed to develop and validate a machine learning model to predict 3-month unfavorable functional outcome after IVT in AIS patients.

**Methods:**

This retrospective study developed ML prognostic models for 3-month functional outcome (modified Rankin scale scores of 3–6) in IVT-treated AIS patients. A derivation cohort (*n* = 938) was split 7:3 for training/testing, with an independent external validation cohort (*n* = 324). The least absolute shrinkage and selection operator (LASSO) regression selected predictors from clinical/neuroimaging/laboratory variables. Eight ML algorithms (including Logistic Regression, Random Forest, Extreme Gradient Boosting, Multilayer Perceptron, Support Vector Machine, Light Gradient Boosting Machine, Decision Tree, and K-Nearest Neighbors) were trained using 10-fold cross-validation and evaluated on test/external sets via the area under the curve (AUC), accuracy, precision, recall and F1-score. Additionally, the SHapley Additive exPlanations (SHAP) interpreted the optimal model.

**Results:**

938 patients constituted the derivation cohort (training: *n* = 656, test: *n* = 282) and 324 patients the external validation cohort. Unfavorable 3-month outcomes (mRS 3–6) occurred in 25.7% and 22.8%, respectively. LASSO regression selected five predictors: the neutrophil-to-lymphocyte ratio (NLR), admission National Institutes of Health Stroke Scale (NIHSS) score, the Alberta Stroke Program Early CT Score (ASPECTS), atrial fibrillation, and blood glucose. While tree-based methods like XGBoost and LightGBM showed elevated training performance (e.g., XGBoost training AUC = 0.878) but significant drops in validation (AUC = 0.791), LR demonstrated optimal performance: robust training AUC (0.792), minimal validation degradation (AUC = 0.787). LR model was subsequently employed as classification method demonstrating optimal performance with (AUC = 0.777) in the test dataset. External validation confirmed LR’s stability (AUC = 0.797). SHAP analysis ranked NLR as the strongest predictor (followed by NIHSS/ASPECTS), with higher values increasing risk. Learning curves indicated no overfitting. A nomogram enabled individualized risk quantification.

**Conclusion:**

A parsimonious 5-variable LR model robustly predicts 3-month post-IVT outcomes, combining clinical utility, interpretability, and generalizability. NLR-driven inflammation is critical to prognosis. This tool facilitates early high-risk patient identification for personalized intervention.

## 1 Introduction

Stroke remains as a global health crisis, ranking as the second leading contributor to mortality worldwide and the third leading cause of long-term disability (1). It imposes a substantial global health burden at both individual and societal levels, with the rate of disability burden increasing more rapidly in low-income and middle-income countries than in high-income countries (2–4). Acute ischemic stroke (AIS) is defined as sudden neurological dysfunction caused by focal brain ischemia lasting more than 24 h or accompanied by evidence of acute infarction on brain imaging, regardless of symptom duration, accounts for approximately 70% of incident stroke events (5, 6). Intravenous thrombolysis (IVT), administered within the 4.5-h time window, constitutes the gold-standard therapy for AIS, as universally endorsed by international guidelines (7). Despite advancements in endovascular thrombectomy, IVT remains the most accessible and efficacious reperfusion treatment for patients with AIS in clinical practice, owing to its widespread availability and relative simplicity of administration (8, 9). Despite its established efficacy in enhancing functional recovery, nearly half of IVT-treated patients experience unfavorable functional outcomes at 3 months. The modified Rankin Scale (mRS; range 0–6, where 6 indicates death), which integrates both motor and cognitive components and encompasses the constructs of impairment, disability, and handicap, is considered to be the most accepted outcome for assessing the efficacy of interventions of AIS (10, 11). Given the substantial neurological disability burden associated with AIS (12), developing validated predictive tools remains imperative for the early identification of patients susceptible to adverse functional outcomes. Such prognostic stratification would facilitate targeted interventions and optimized resource allocation, ultimately improving long-term neurological prognosis. However, many existing prediction models are limited by their suboptimal predictive accuracy and the lack of robust external validation, resulting in uncertain generalizability to broader, more diverse populations (13, 14). Furthermore, numerous tools rely on high-dimensional data—incorporating extensive imaging, genomic, or biomarker variables—which complicates clinical interpretation and practical implementation, thereby hindering widespread adoption (15, 16). The development of novel, concise, yet robust prediction tools is therefore essential to enhance clinical relevance and facilitate translation into routine care.

Inflammation and immune responses critically mediate all phases of cerebral ischemia pathogenesis. Following ischemic insult, the inflammatory response initiated promptly. Focal brain ischemia stimulates what is called sterile inflammation (17), trigger inflammatory signaling through the activation of microglia, which subsequently release pro-inflammatory cytokines and chemokines, thereby promoting robust pro-inflammatory cascades, propelling the pathophysiological progression (18, 19). Critically, ischemic microenvironments trigger local immune responses, characterized by inflammatory cytokine production, which exacerbate blood–brain barrier (BBB) permeability (20, 21). Notably, neutrophils are the earliest leukocytes recruited from peripheral blood into the brain (22, 23). Neutrophils induce neurotoxicity through multiple mechanisms such as the participation in thrombus formation and expansion, upregulation of matrix metalloproteinases, excessive generation of reactive oxygen species, and the release of neutrophil extracellular traps (NETs) (24–26). The subsequent increase in capillary permeability, disruption of the BBB, and cellular edema can collectively impair post-stroke revascularization and vascular remodeling, thereby adversely affecting stroke outcomes (27). Clinical studies demonstrated the early increase of peripheral neutrophils as an independent predictor of neurological deterioration and poor outcome (28, 29). In addition, acute central nervous system injury can induce a state of immunodepression by activating the sympathetic nervous system and hypothalamic–pituitary–adrenal axis, leading to elevated catecholamines and steroids that cause apoptosis and functional deactivation of peripheral lymphocytes (30). Lymphocytes serve as pivotal regulators of host defense, and their depletion markedly elevates susceptibility to infections. Clinical research data indicates that low lymphocyte counts constitute an independent predictor of infection risk in stroke patients (31, 32). Emerging evidence underscores the prognostic significance of these mechanisms of leukocyte-derived inflammation in post-stroke outcomes (27), with the neutrophil-to-lymphocyte ratio (NLR) validated as a predictive biomarker for clinical outcome in AIS patients receiving IVT (33). While baseline NLR has been established as an independent risk factor for outcomes including early neurological improvement (ENI), hemorrhagic transformation (HT), and mortality in AIS patients (34), the predominant focus of current NLR research on univariate assessments fails to capture synergistic interactions with clinical covariates (35). This methodological constraint impedes clinical translation, given that isolated biomarkers inherently lack the discriminative power for complex multifactorial outcomes.

Machine learning (ML), a rapidly advancing branch of artificial intelligence (AI), leveraging computational advances to uncover predictive insights from high-dimensional data, demonstrates growing utility in clinical stroke research (36, 37). ML offers substantial advantages in predictive accuracy and in identifying previously overlooked patient subgroups defined by unique physiological characteristics and prognostic trajectories. Various methodologies exist for feature selection within the domain of ML. Notably, the least absolute shrinkage and selection operator (LASSO) regression distinguishes itself from conventional stepwise regression techniques, which utilize forward or backward variable selection, by facilitating the effective screening of a greater number of variables even when the sample size is limited (38). Moreover, LASSO regression provides superior feature selection from high-dimensional biomedical datasets while addressing multicollinearity limitations inherent in conventional methods (39). As a result, LASSO-based ML methods demonstrate enhanced prognostic discrimination across diverse medical applications (40–42). Furthermore, to compensate for the scarcity of interpretable evidence supporting predictive models, we deployed the SHapley Additive exPlanations (SHAP) analysis. This technique offers intuitive, feature-level explanations, which are critical for validating model efficacy and building trust (43). Consequently, integrating complementary clinical variables using ML models and SHAP interpretation may optimize the prediction of unfavorable outcomes for post-IVT AIS patients.

Therefore, we aimed to develop and validate a machine learning model for predicting 3-month functional outcomes in IVT-treated AIS patients, incorporating interpretability analysis to elucidate predictor contributions to the model predictions.

## 2 Materials and methods

### 2.1 Study population

This retrospective study enrolled patients diagnosed with AIS who received IVT within the 4.5-h treatment window. The derivation cohort consisted of 938 patients treated at The Affiliated Hospital of Xuzhou Medical University between September 2020 and October 2024. Admission non-contrast head computed tomography (CT) confirmed the absence of acute hemorrhage. An independent external validation cohort comprised 324 consecutive patients treated with IVT for AIS at Hongze District People’s Hospital between January 2019 and December 2022. Identical inclusion and exclusion criteria were applied to both cohorts. Inclusion criteria were: (1) over 18 years of age; (2) clinically and neuroimaging-confirmed diagnosis of AIS; (3) within 4.5 h of symptom onset, followed by recombinant tissue plasminogen activator (rt-PA) treatment (0.9 mg/kg up to a maximum of 90 mg, 10% of the dose as a bolus followed by a 60-min infusion of the remaining dose). Exclusion criteria were: (1) pre-stroke modified Rankin Scale (mRS) scores > 2, indicating significant pre-existing disability; (2) unavailable 3-month post-stroke mRS assessment; (3) receipt of subsequent endovascular thrombectomy; (4) active malignancy or major trauma at admission; (5) incomplete clinical data. To assess potential selection bias, we compared patients excluded due to missing data with the final derivation cohort across baseline characteristics. The study protocol received approval from the Ethics Committee of The Affiliated Hospital of Xuzhou Medical University (Approval number: XYFY2025-KL044-01). Given its retrospective design using anonymized data, the requirement for written informed consent was waived.

### 2.2 Data collection

The analysis incorporated these clinical variables: (1) Demographics: age, sex and body mass index (BMI). (2) Medical history: hypertension, diabetes mellitus (DM), coronary heart disease (CHD), atrial fibrillation (AF), anticoagulant/antiplatelet medications, smoking status, and alcohol consumption; (3) Clinical features: admission systolic/diastolic blood pressure (SBP/DBP), onset-to-treatment time (OTT), National Institutes of Health Stroke Scale (NIHSS) score at admission and discharge, Trial of ORG 10172 in Acute Stroke Treatment (TOAST) classification, Alberta Stroke Program Early CT Score (ASPECTS) (44), mRS score at 3 months; (4) Laboratory indices: admission levels of neutrophil, lymphocyte, platelet, eosinophil counts, blood glucose, albumin, and glycated hemoglobin (HbA1c). The neutrophil-to-lymphocyte ratio (NLR) was calculated at admission by dividing absolute neutrophil count by absolute lymphocyte count.

### 2.3 Outcome definition

Functional outcome was assessed using the mRS 3 months after IVT. Evaluations were performed during scheduled clinic visits by board-certified neurologists blinded to the predictive model development. For patients unable to attend clinic, structured telephone interviews were conducted by trained research nurses using a validated protocol to ensure reliable mRS scoring. The primary outcome was unfavorable functional outcome, defined as mRS score 3–6. A favorable outcome was defined as mRS score 0–2 (45).

### 2.4 Feature selection

Feature selection was performed using the Least Absolute Shrinkage and Selection Operator (LASSO) regression (46). This regularization technique minimizes overfitting by applying an L1 penalty term that shrinks coefficients and drives some coefficients of non-informative features to zero. Continuous variables were standardized (mean = 0, standard deviation = 1) before model fitting to ensure equivalent scaling of the penalty term. LASSO regression was performed on the derivation cohort training set (70% of derivation cohort). Feature subset optimization against overfitting was achieved by determining the optimal regularization parameter (*λ*) value through the standard error of the minimum distance based 10-fold cross-validation (47). Features with non-zero coefficients after LASSO regularization were retained for subsequent modeling.

### 2.5 Machine learning model development and evaluation

#### 2.5.1 Model development

Eight supervised machine learning algorithms were trained to predict the 3-month unfavorable functional outcome using the features selected by LASSO: Logistic Regression (LR), Random Forest (RF), Extreme Gradient Boosting (XGBoost), Multilayer Perceptron (MLP), Support Vector Machine (SVM), Light Gradient Boosting Machine (LightGBM), Decision Tree (DT), and K-Nearest Neighbors (KNN). Models were implemented using Python libraries (scikit-learn 0.22.1, XGBoost 1.2.1, LightGBM 3.2.1). The derivation cohort was randomly stratified by outcome and split into a training set (70%) and a held-out internal test set (30%). Hyperparameter tuning for each algorithm was performed exclusively on the training set using a nested 10-fold cross-validation strategy. The inner loop of the cross-validation was optimized by maximizing the Area Under the Receiver Operating Characteristic Curve (ROC-AUC). The internal test set was used only once for the final comparative evaluation of all tuned models.

We implemented a comprehensive tuning strategy using grid search with cross-validation. For tree-based models (XGBoost, LightGBM, Random Forest, Decision Tree), we focused on regularization parameters including max_depth, min_samples_split, and reg_lambda to control model complexity and prevent overfitting. For linear models (Logistic Regression, SVM), we optimized regularization strength through the C parameter. All preprocessing steps were fitted solely on the training folds of the inner loop to prevent any data leakage. The optimized hyperparameters from the inner loop were then used to train a final model on the entire training set for evaluation on the held-out internal test set.

#### 2.5.2 Model evaluation and comparison

Model performance was assessed using: (1) Discrimination: Primary metric: Area Under the Receiver Operating Characteristic Curve (ROC-AUC). Secondary metrics: Accuracy, Precision, Recall, F1-Score. Optimal classification thresholds were determined by maximizing the Youden Index on the validation folds. ROC curves and AUC values were generated for all datasets: internal training (using cross-validation predictions), internal test set, and external validation set. (2) Calibration: Calibration curves plotted predicted probabilities against observed event frequencies (Python, sklearn 0.22.1). Perfect calibration demonstrates along the 45° line. The Brier score was also reported (lower score indicates better calibration, range 0–1). (3) Clinical Utility: Decision Curve Analysis (DCA) implemented in R software (rmda 1.6) assessed the net benefit across a range of probability thresholds (15–35%) relevant for clinical decision-making. The performance metrics on the internal test set were compared across all eight algorithms to identify the optimal predictive model.

#### 2.5.3 Model interpretation

The SHapley Additive exPlanations (SHAP) method (Python SHAP v0.39.0) was applied to the selected optimal model for interpretability (48). SHAP values attribute a contribution value to each feature for each individual prediction, enabling local and global interpretability. Graphical depiction techniques included: (1) Summary plots identifying the five most influential covariates through value magnitude visualization; (2) Dependency plots elucidating marginal effect relationships between feature variations and Shapley value fluctuations; (3) Global feature importance analysis combined with partial dependence evaluations. This integrated approach delineates directional associations between explanatory variables and adverse outcome predictions.

#### 2.5.4 External validation

The generalizability of the final optimized model was evaluated by applying the parameters trained on the full derivation cohort training set to the independent, prospectively collected external validation cohort from Hongze District People’s Hospital. AUC, sensitivity and specificity were computed.

### 2.6 Statistical analysis

Statistical analysis was conducted using SPSS (Statistical Package for the Social Sciences, v26.0), R (v4.2.3), and Python (v3.11.4). Continuous variables were summarized as mean ± standard deviation (SD) or medians (IQRs), with group comparisons conducted using the Mann–Whitney U test. Categorical variables were reported as frequency percentages (%), analyzed through Pearson’s *χ*^2^ or Fisher’s exact tests. Independent predictor capacity was expressed through odds ratios (95% confidence intervals). All statistical tests were two-tailed, adopting *p*-value < 0.05 as the significance statistically.

## 3 Results

### 3.1 Baseline characteristics

**Figure 1:**
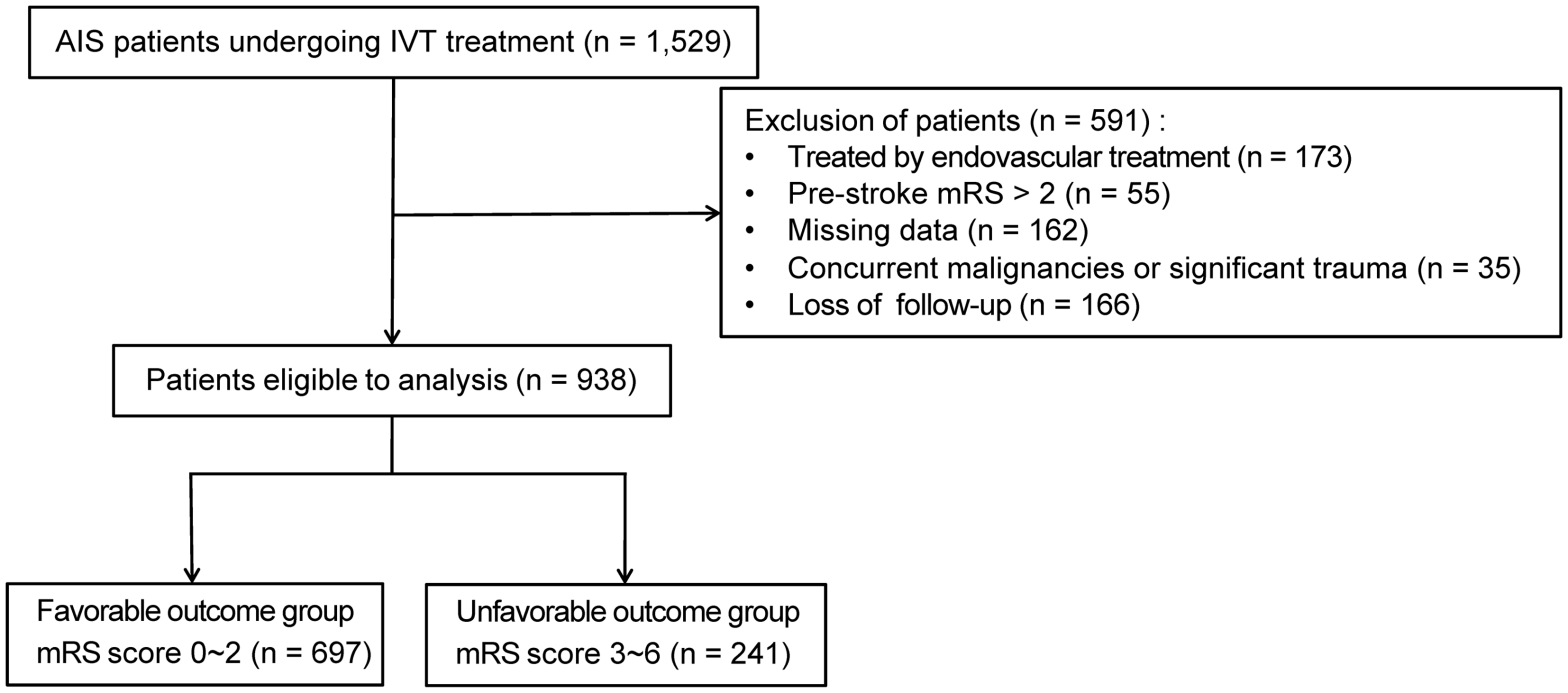
Flowchart of patient selection. AIS, acute ischemic stroke; IVT, intravenous thrombolysis; mRS score, modified Rankin Scale.

This research initially enrolled 1,529 patients diagnosed with AIS and received IVT within 4.5 h of symptom onset. After applying the exclusion criteria, the final derivation cohort consisted of 938 patients (Figure 1). Exclusions included: endovascular therapy (*n* = 173), pre-stroke mRS > 2 (*n* = 55), missing data (*n* = 162), concurrent malignancy or major trauma (*n* = 35), and loss to 3-month follow-up (*n* = 166). Patients missing essential record required for model development (*n* = 162) were excluded. To assess potential selection bias, we compared these excluded patients with the included derivation cohort (*n* = 938) across baseline characteristics including demographics, clinical features, and laboratory indices. No significant differences were observed in any variable (all *p*-values > 0.05; Supplementary Table 1), indicating comparable profiles between groups. This supports the representativeness of the analyzed cohort despite missing data handling via complete-case analysis. The derivation cohort was randomly split into a training set (70%, *n* = 656) and an internal test set (30%, *n* = 282). Baseline characteristics did not differ significantly (*p* > 0.05) between the training and internal test sets (Supplementary Table 2), confirming successful randomization and mitigating selection bias.

The overall derivation cohort (*n* = 938) had a mean age of 68 years (range 59–77), with males comprising 65.9%. Table 1 details clinical profiles stratified by 3-month functional outcome (favorable with mRS 0–2 vs. unfavorable with mRS 3–6). Unfavorable outcomes occurred in 241 patients (25.7%). The external validation set (*n* = 324) showed a comparable unfavorable outcome rate of 22.8% (*n* = 74). Reduced functional recovery significantly correlated with multiple clinical indicators including: advanced age, atrial fibrillation, smoking/drinking history, anticoagulant therapy, admission SBP, onset-to-treatment time, ASPECTS, baseline NIHSS, TOAST classification, NLR, platelet count, eosinophil level, albumin, RDW, HDL, and glucose levels (*p* < 0.05; Table 1).

**Table 1:** Baseline characteristics of the subgroup according to clinical outcomes.

Variables	Total (*n* = 938)	Favorable outcome group (*n* = 697)	Unfavorable outcome group (*n* = 241)	*p* value
Demographics				
Age, median (IQR)	68 (59, 77)	68 (59, 76)	70 (60, 79)	0.010
Gender, (male, %)	618 (65.88)	465 (66.71)	153 (63.49)	0.362
BMI, median (IQR)	24.80 (22.85, 27.06)	24.82 (22.86, 27.06)	24.77 (22.49, 27.06)	0.580
Previous history				
Hypertension, *n* (%)	605 (64.50)	450 (64.56)	155 (64.32)	0.945
DM, *n* (%)	218 (23.24)	160 (22.96)	58 (24.07)	0.725
CHD, *n* (%)	149 (15.88)	107 (15.35)	42 (17.43)	0.447
AF, *n* (%)	97 (10.34)	51 (7.32)	46 (19.09)	<0.001
Previous stroke, *n* (%)	275 (29.32)	197 (28.26)	78 (32.37)	0.228
Anticoagulant therapy, *n* (%)	135 (14.39)	79 (11.33)	56 (23.24)	<0.001
Smoking, *n* (%)	328 (34.97)	266 (38.16)	62 (25.73)	<0.001
Drinking, *n* (%)	153 (16.31)	127 (18.22)	26 (10.79)	0.007
Baseline parameters				
SBP, median (IQR)	151 (138, 165)	150 (137, 164)	155 (140, 168)	0.020
DBP, median (IQR)	86 (78, 94)	85 (78, 94)	87 (77, 97)	0.409
OTT, median (IQR)	185 (130, 237)	175 (125, 232)	205 (150, 253)	<0.001
ASPECTS, median (IQR)	8 (7, 8)	8 (7, 9)	7 (6, 8)	<0.001
Baseline NIHSS score, median (IQR)	6 (4, 11)	6 (4, 8)	10 (6, 17)	<0.001
NIHSS score after IVT, median (IQR)	3 (2, 8)	3 (1, 6)	9 (4, 15)	<0.001
TOAST classification				<0.001
Large-artery atherosclerosis, *n* (%)	636 (67.80)	471 (67.58)	165 (68.46)	
Cardioembolic, *n* (%)	114 (12.15)	70 (10.04)	44 (18.26)	
Small-artery occlusion, *n* (%)	181 (19.30)	149 (21.38)	32 (13.28)	
Other etiology, *n* (%)	6 (0.64)	6 (0.86)	0 (0.00)	
Undetermined etiology, *n* (%)	1 (0.11)	1 (0.14)	0 (0.00)	
Laboratory data				
Neutrophil, median (IQR)	4.88 (3.63, 6.47)	4.57 (3.47, 5.92)	6.02 (4.47, 8.39)	<0.001
Lymphocyte, median (IQR)	1.6 (1.2, 2.2)	1.7 (1.3, 2.3)	1.4 (0.9, 1.8)	<0.001
NLR, median (IQR)	2.94 (1.95, 4.83)	2.63 (1.79, 4.01)	4.65 (2.64, 7.79)	<0.001
Platelets, median (IQR)	199 (162, 235)	202 (165, 236)	185 (157, 231)	0.025
Eosinophils, median (IQR)	0.08 (0.04, 0.15)	0.10 (0.05, 0.16)	0.05 (0.02, 0.11)	<0.001
Albumin, median (IQR)	42.2 (39.2, 44.9)	42.3 (39.7, 44.9)	41.6 (38.0, 44.7)	0.011
Hemoglobin, median (IQR)	141 (130, 152)	141 (131, 152)	140 (127, 152)	0.249
RDW, median (IQR)	12.9 (12.4, 13.4)	12.9 (12.4, 13.3)	12.9 (12.6, 13.5)	0.049
TC, median (IQR)	4.48 (3.91, 5.01)	4.48 (3.89, 5.04)	4.46 (3.97, 4.93)	0.974
TG, median (IQR)	1.36 (0.97, 1.64)	1.35 (0.98, 1.65)	1.36 (0.95, 1.58)	0.490
HDL, median (IQR)	1.07 (0.91, 1.17)	1.05 (0.90, 1.17)	1.07 (0.92, 1.17)	0.041
LDL, median (IQR)	2.56 (2.12, 3.00)	2.56 (2.07, 3.01)	2.56 (2.20, 3.00)	0.129
CRP, median (IQR)	1.55 (0.60, 4.50)	1.50 (0.60, 4.30)	1.80 (0.60, 5.00)	0.183
UA, median (IQR)	308 (255, 366)	305 (255, 366)	311 (249, 363)	0.936
AST, median (IQR)	24.00 (20.00, 29.00)	24.00 (20.00, 30.00)	25.00 (20.00, 29.00)	0.721
ALT, median (IQR)	20.00 (15.00, 29.00)	20.00 (15.00, 29.00)	20.00 (15.00, 29.00)	0.826
GGT, median (IQR)	23.00 (15.00, 35.00)	22.00 (15.00, 35.00)	23.00 (16.00, 35.00)	0.658
HbA1c, median (IQR)	6.08 (5.60, 6.65)	6.00 (5.60, 6.50)	6.30 (5.70, 6.65)	0.077
Blood glucose, median (IQR)	5.65 (4.90, 7.43)	5.48 (4.84, 6.99)	6.20 (5.15, 7.90)	<0.001
Treatment after admission				
Hemorrhagic transformation, *n* (%)	112 (11.94)	33 (4.73)	79 (32.78)	<0.001
sICH, *n* (%)	51 (5.44)	5 (0.72)	46 (19.09)	<0.001
NIHSS score on discharge, median (IQR)	2 (0, 6.00)	1 (0, 3)	13 (7, 25)	<0.001

BMI, Body Mass Index; DM, diabetes mellitus; AF, Atrial fibrillation; CHD, coronary heart disease; OTT, Onset-to-treatment; NIHSS, National Institutes of Health Stroke Scale; SBP, systolic blood pressure; DBP, diastolic blood pressure; ASPECTS, Alberta Stroke Program Early CT Score; TOAST, Trial of ORG 10172 in Acute Stroke Treatment; IVT, intravenous thrombolysis; RDW, red cell distribution width; LDL, low-density lipoprotein; HDL, high-density lipoprotein; NLR, neutrophil-to-lymphocyte ratio; HT, hemorrhagic transformation; sICH, Symptomatic Hemorrhagic transformation.

### 3.2 Feature selection for outcome prediction

**Figure 2:**
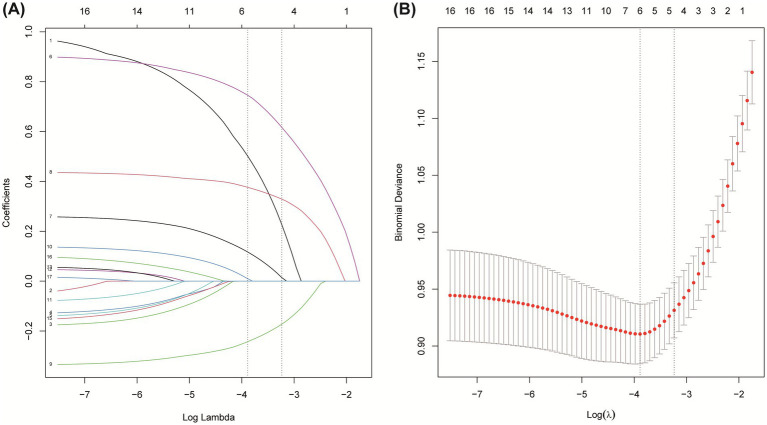
Feature selection based on LASSO model. **(A)** Coefficient profiles during LASSO regularization. **(B)** Ten-fold cross-validation for identifying the optimal *λ* value.

The selection of predictive features was performed using Least Absolute Shrinkage and Selection Operator (LASSO) regression, a penalized regression technique designed to handle multicollinearity and prevent overfitting by shrinking the coefficients of non-informative variables to zero. An initial pool of 17 clinically accessible variables, encompassing demographics, medical history, clinical presentation, imaging features, and laboratory indices, was standardized and entered into the model. The optimal regularization parameter (*λ*) was determined via 10-fold cross-validation on the derivation training set (*n* = 656), minimizing the binomial deviance. This process identified the optimal λ parameter (lambda with minimum distance = 0.039; Figure 2), which addresses multicollinearity and overfitting through coefficient shrinkage (49). At this optimal λ, the model retained five variables with non-zero coefficients: NLR, baseline NIHSS, ASPECTS, atrial fibrillation, and blood glucose. Subsequently, multivariate logistic regression analysis confirmed that each of these five variables was independently associated with an increased risk of unfavorable outcome (*p* < 0.05; Table 2) (50). Odds ratios (OR) with 95% confidence intervals (CI) are reported in Table 2, and the LASSO coefficients are provided in Supplementary Table 3.

**Table 2:** Multivariate logistic regression analysis.

Variable	Estimate	SE	*Z*	*p*	OR (95% CI)
(Intercept)	−1.553	0.683	−2.274	0.023	0.212 (0.055–0.798)
NLR	0.294	0.031	9.345	<0.001	1.342 (1.264–1.430)
NIHSS	0.06	0.012	5.187	<0.001	1.062 (1.038–1.087)
ASPECTS	−0.287	0.078	−3.684	<0.001	0.751 (0.644–0.874)
Atrial fibrillation	0.828	0.254	3.261	0.001	2.288 (1.386–3.756)
Blood glucose	0.098	0.033	2.943	0.003	1.103 (1.034–1.178)

SE, Standard error; OR, odds ratio; CI, confidence interval; NLR, neutrophil-to-lymphocyte ratio; NIHSS, National Institutes of Health Stroke Scale; ASPECTS, Alberta Stroke Program Early CT Score.

### 3.3 Models performance comparisons

Eight machine learning models (XGBoost, LR, LightGBM, RF, DT, MLP, SVM, KNN) were trained and tuned using the five selected predictors on the derivation training set via 10-fold cross-validation. Performance was independently evaluated on the held-out internal test set. Our optimization encompassed tuning of core hyperparameters across all model types. For tree-based models (e.g., XGBoost, LightGBM), we focused on parameters controlling tree complexity (e.g., depth constraints, leaf node quantities), regularization intensity (e.g., L1/L2 coefficients), and key overfitting prevention mechanisms. For linear models (e.g., Logistic Regression, SVM), we systematically optimized regularization types and strengths. All parameters were determined through comprehensive grid search with cross-validation.

**Figure 3:**
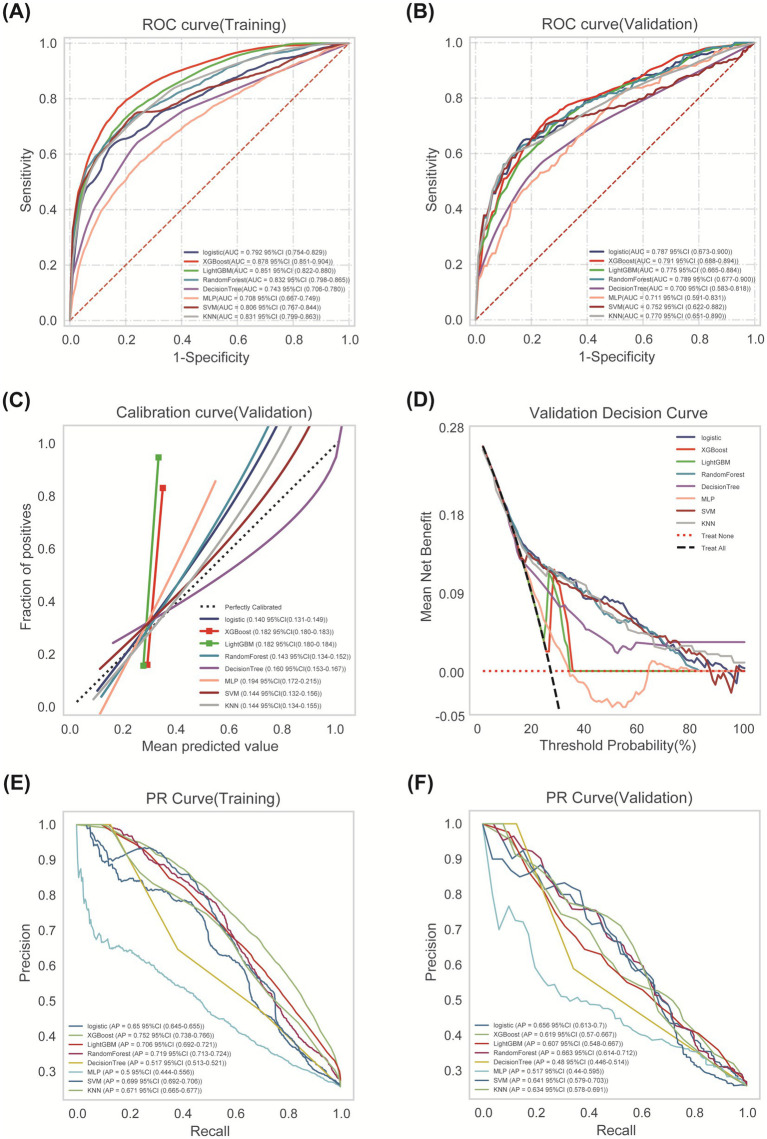
Comprehensive analysis of ML algorithms. **(A)** ROC curves comparing discrimination performance of eight ML models for predicting post-thrombolysis outcomes in the training cohort. **(B)** ROC curves demonstrating generalization performance in validation cohort. **(C)** Calibration curves depicting agreement between predicted probabilities (x-axis) and observed event frequencies (y-axis). Logistic Regression showed optimal calibration. **(D)** Decision Curve Analysis (DCA) assessing net benefit across probability thresholds. **(E)** Training set PR curve. **(F)** Test set PR curve. Precision-recall relationships at varying probability thresholds. Horizontal dashed line indicates positive event rate.

Comprehensive performance evaluation revealed distinct characteristics among the models. As shown in Figure 3A, all models demonstrated reasonable training performance without perfect discrimination (AUC range: 0.708–0.878), indicating successful mitigation of overfitting through our optimized regularization approach. While tree-based methods like XGBoost and LightGBM showed elevated training performance (e.g., XGBoost training AUC = 0.878; Figure 3A) but significant drops in validation (AUC = 0.791; Figure 3B), Logistic Regression achieved stable cross-validated performance (AUC = 0.792, 95% CI: 0.754–0.829) that was maintained on the validation set (AUC = 0.787, 95% CI: 0.673–0.900) with minimal performance degradation (ΔAUC = 0.005) (Figure 3B). Critical assessment of model calibration demonstrated LR’s superior reliability with optimal Brier score (0.140, 95% CI: 0.131–0.149), significantly outperforming other models in calibration accuracy (Figure 3C). Decision Curve Analysis confirmed LR’s clinical utility, providing the greatest net benefit across clinically relevant threshold probabilities (15–35%) (Figure 3D). Precision-Recall analysis further supported LR’s robust performance (AP = 0.656) in handling class imbalance (Figures 3E,F). Accuracy, Precision, Recall, F1-Score and Cutoff value for all models are detailed in Table 3. Collectively, based on its optimal balance of discriminatory ability, calibration reliability, and clinical utility across multiple metrics, Logistic Regression was selected as the preferred model for deployment.

**Table 3:** Predictive performance metrics of different ML models in the validation set.

Models	Validation set							
	LR	XGBoost	LightGBM	RF	DT	MLP	SVM	KNN
AUC (95%CI)	0.787 (0.673–0.900)	0.791 (0.688–0.894)	0.775 (0.665–0.884)	0.789 (0.677–0.900)	0.700 (0.583–0.818)	0.711 (0.591–0.831)	0.752 (0.622–0.882)	0.770 (0.651–0.890)
Cutoff value	0.319	0.274	0.258	0.297	0.354	0.385	0.162	0.285
Accuracy (95%CI)	0.791 (0.767–0.815)	0.757 (0.728–0.786)	0.738 (0.708–0.768)	0.771 (0.744–0.798)	0.685 (0.637–0.734)	0.677 (0.626–0.728)	0.722 (0.688–0.756)	0.750 (0.712–0.789)
Precision (95%CI)	0.594 (0.547–0.641)	0.519 (0.478–0.560)	0.495 (0.455–0.535)	0.554 (0.507–0.601)	0.439 (0.386–0.493)	0.424 (0.363–0.485)	0.477 (0.433–0.520)	0.536 (0.459–0.614)
Recall (95%CI)	0.627 (0.576–0.677)	0.677 (0.589–0.764)	0.647 (0.542–0.753)	0.627 (0.556–0.698)	0.639 (0.574–0.705)	0.606 (0.516–0.697)	0.693 (0.649–0.737)	0.614 (0.553–0.676)
F1 score (95%CI)	0.607 (0.568–0.645)	0.585 (0.530–0.641)	0.553 (0.497–0.609)	0.583 (0.538–0.627)	0.514 (0.469–0.559)	0.492 (0.432–0.552)	0.563 (0.522–0.605)	0.562 (0519–0.605)

LR, Logistic Regression; RF, Random Forest; XGBoost, Extreme Gradient Boosting; MLP, Multilayer Perceptron; SVM, Support Vector Machine; LightGBM, Light Gradient Boosting Machine; DT, Decision Tree; KNN, K-Nearest Neighbors.

### 3.4 Development and validation of the optimal model

**Figure 4:**
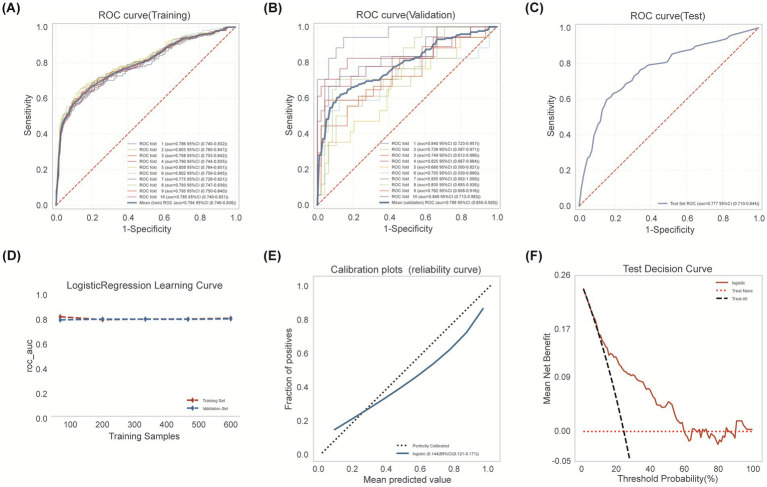
Logistic regression model development encompassed training cohort modeling, cross-validation refinement, and independent testing cohort verification. **(A)** Training set ROC analysis with 10-fold cross-validation. **(B)** Validation set ROC performance across 10 folds. **(C)** Test set discrimination performance (AUC 0.777, 95% CI: 0.710–0.844). Blue: model performance; red dashed: random classifier. **(D)** Learning curve: AUC by training sample size. Validation (blue dashed) converges with training (red) beyond 300 samples, indicating minimal overfitting. **(E)** Calibration curve showing agreement between predicted probabilities and observed outcomes. **(F)** Decision curve analysis: Logistic model (red solid) shows superior net benefit versus treat-all (black dashed) and treat-none (red dotted) strategies, particularly at 20–50% thresholds. Peak net benefit (0.17) occurs at 30% risk threshold.

The optimal Logistic Regression model, using the five selected predictors (AF, ASPECTS, NIHSS, Blood Glucose, NLR), was refined on the entire derivation training set (*n* = 656) using 10-fold cross-validation. The mean cross-validated AUC was 0.794 (95% CI: 0.749–0.838; range across folds: 0.728–0.851; Figure 4A). Validation set performance maintained stability (mean AUC 0.788, 95% CI: 0.655–0.920) (Figure 4B). Evaluation on the internal test set (*n* = 282) demonstrated sustained performance (AUC = 0.777, 95% CI: 0.710–0.844; Figure 4C). Accuracy, specificity, and sensitivity on the test set were 0.791, 0.860, and 0.567, respectively. The learning curve analysis indicated stable model performance, with converging training and validation accuracy estimates remaining above 80% without significant divergence as the sample size increased, suggesting the model was adequately fitted without overfitting (Figure 4D). Calibration on the test set was moderate (Figure 4E; Brier Score = 0.140). Decision Curve Analysis confirmed positive net benefit across the same relevant probability thresholds (Figure 4F).

### 3.5 Optimal model interpretation

**Figure 5:**
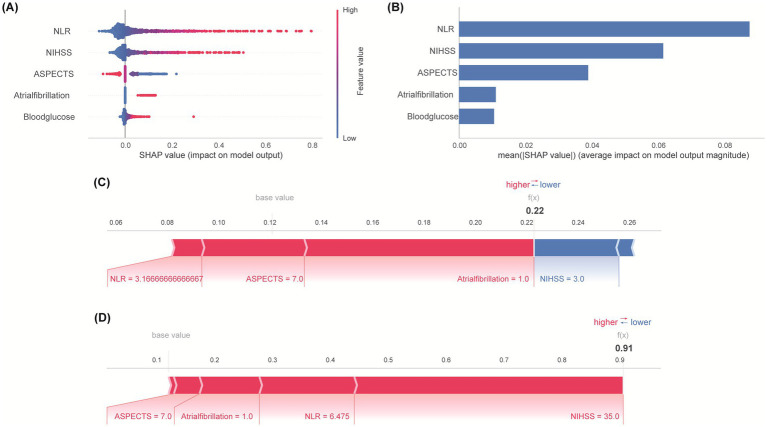
Feature importance and interpretation. **(A)** SHAP summary plot: Directional impact of predictors on outcome risk. Point position indicates effect direction—positive values denote enhanced risk probability, while negative counterparts indicate protective functions. **(B)** Quantitative comparison of predictive influence magnitude. Error bars represent standard error of mean absolute SHAP values. **(C)** SHAP explanation for a patient with actual favorable outcome. **(D)** SHAP explanation for a patient with actual unfavorable outcome. Arrow length encodes predictor influence magnitude on prediction.

SHapley Additive exPlanations (SHAP) analysis was employed to interpret the final Logistic Regression model globally and locally. The SHAP summary plot (Figure 5A) illustrates the impact and directionality of each predictor. Higher NLR values and NIHSS scores consistently increased the risk of an unfavorable outcome, while higher ASPECTS scores decreased the risk. Presence of AF and higher blood glucose levels also generally increased the risk. NLR exhibited predominant risk-enhancing properties, where elevated values substantially increasing adverse outcome risk. Mean absolute SHAP value analysis ranked the features by their overall contribution to the model’s predictions: NLR was the most influential predictor, followed by NIHSS, ASPECTS, Atrial Fibrillation and Blood Glucose (Figure 5B). Examples of local interpretability are shown in Figure 5C (patient predicted low risk, actual favorable outcome) and Figure 5D (patient predicted high risk, actual unfavorable outcome). Each feature’s specific value and its SHAP contribution (increasing or decreasing the predicted probability away from the base value) combine to generate the individual prediction.

### 3.6 External validation and clinical application

**Figure 6:**
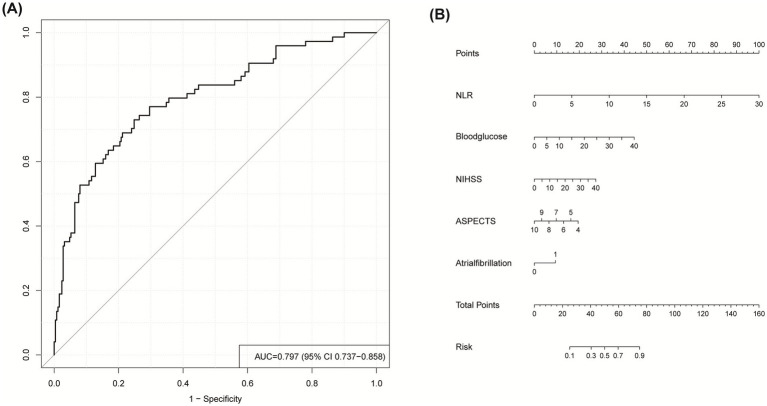
**(A)** ROC curve for predicting unfavorable outcome in external validation cohort. **(B)** Nomogram for clinical risk prediction: Point-based scoring system. To estimate probability: (1) Locate predictor value on corresponding axis; (2) Draw vertical line to Points axis; (3) Sum points; (4) Project total to probability axis.

External validation was conducted using an independent external Hongze District People’s Hospital validation cohort comprising 324 stroke patients receiving IVT, with unfavorable outcomes documented in 74 cases (22.8%). It demonstrated robust generalizability, achieving an AUC of 0.797 (95% CI: 0.737–0.858; Figure 6A), sensitivity of 0.730, and specificity of 0.752. To facilitate clinical implementation, a nomogram was constructed based on the final logistic regression coefficients (Figure 6B), enabling clinicians to estimate individualized probabilities of an unfavorable 3-month outcome for patients receiving IVT for AIS.

## 4 Discussion

This study has developed and validated a machine learning-based predictive model for 3-month functional outcomes following IVT in AIS patients. Using LASSO regression for feature selection followed by multivariate logistic regression, we identified five key predictors from 17 candidate variables (51): NLR, baseline NIHSS, ASPECTS, atrial fibrillation, and blood glucose. This parsimonious set of readily available clinical, radiological, and inflammatory biomarkers provides a practical foundation for prognostic assessment. We comprehensively evaluated eight machine learning models: Logistic Regression (LR), Random Forest (RF), Extreme Gradient Boosting (XGBoost), Multilayer Perceptron (MLP), Support Vector Machine (SVM), Light Gradient Boosting Machine (LightGBM), Decision Tree (DT), and K-Nearest Neighbors (KNN) (52). Based on the comprehensive performance evaluation across all validation metrics, logistic regression emerged as the most stable and reliable model, consistently demonstrating balanced performance without signs of overfitting. In contrast to complex tree-based ensemble methods (e.g., XGBoost, LightGBM) and other non-linear algorithms, which exhibited enhanced in-sample metrics (e.g., XGBoost training AUC = 0.878) yet notable declines in external validation (AUC = 0.791), logistic regression preserved strong discriminative capability on the training set (AUC = 0.792) and validation set (AUC = 0.787) with only marginal deterioration. A critical advantage of LR was its exceptional calibration precision, reflected by a Brier score of 0.140, indicating close agreement between forecast probabilities and actual event rates. Moreover, it yielded the greatest and most consistent net clinical benefit throughout the spectrum of relevant probability thresholds as evidenced by decision curve analysis. This stability likely reflects the intrinsic alignment between LR’s linear decision boundary and the predominantly additive relationships among our predictors, whereas unnecessary nonlinear complexity impaired generalization in other models.

The reliability and stability of our final logistic regression model were rigorously interrogated through a comprehensive validation framework. Internally, the model showed consistent performance with minimal degradation from cross-validation (mean AUC = 0.788) to the held-out test set (AUC = 0.777), while calibration metrics revealed excellent agreement between predicted probabilities and observed outcomes (Brier score = 0.144). Externally, the model maintained robust discrimination (AUC = 0.797) in an independent validation cohort (*n* = 324) with consistent sensitivity (0.730) and specificity (0.752). Furthermore, biological plausibility was confirmed through SHAP analysis, which demonstrated consistent risk directionality across cohorts (higher NLR/NIHSS/glucose increasing risk, higher ASPECTS reducing risk), thereby reinforcing model stability.

When compared to existing prognostic models, our approach demonstrates three distinct advantages. First, it exhibits superior generalizability, maintaining robust discrimination (AUC > 0.777) across both internal validation and independent external cohorts—a critical advancement for real-world implementation often lacking in single-center models. Second, it offers unmatched clinical practicality through its parsimonious nature, utilizing only five clinically routinely available variables without requiring specialized tests, complex computations, or additional costs. Third, it provides innovative biological interpretability through SHAP analysis, which not only quantifies feature contributions but also reveals clinically actionable interactions between predictors, enabling personalized risk assessment beyond conventional scoring systems.

SHAP interpretability analysis affirmed the central role of our five predictors. The analysis indicated that higher baseline NIHSS scores indicate heightened initial neurological deficits (53), and higher blood glucose synergistically amplify adverse outcome risks, consistent with cerebrovascular pathogenesis (54). Several mechanisms underlying stress-induced hyperglycemia pathology may explain this: Hepatic overproduction and insulin resistance further impair rt-PA fibrinolysis, compromises blood–brain barrier (BBB) integrity, and aggravate cerebral edema (55, 56). Conversely, diminished ASPECTS, signifying extensive early parenchymal injury (57, 58), and pre-existing atrial fibrillation emerged as independent radiological and comorbid risk factors. Mechanistically, atrial fibrillation promotes cardioembolic clot resistance to lysis during thrombolysis, worsening ischemic injury (59, 60). Crucially, NLR ranked as a principal effector, outperforming conventional biomarkers (61, 62). Substantial evidence confirms that inflammatory mediators critically influence cerebral ischemia pathogenesis (63–65). Following stroke onset, neutrophils constitute the earliest responding immune cells that amplify tissue damage through chemokine release and matrix metalloproteinase-9 (MMP-9) overexpression (26, 61), exacerbating BBB disruption and contributing to hemorrhagic transformation (66). Notably, neutrophil extracellular traps (NETs) demonstrate elevated plasma levels that correlate with poor prognoses (26). Supporting the importance of neurological severity, several studies indicate that baseline NIHSS scores correlate with 3-month functional recovery after IVT (67, 68). Additionally, clinical metrics like NLR independently predict hemorrhagic complications and mortality (61, 62). The use of LR, reinforced by SHAP interpretation, enhances clinical trust and facilitates integration into decision-making workflows.

However, our study also has several limitations. First, biomarkers were measured at a single timepoint, lacking temporal dynamics. Second, despite external validation, model development relied on retrospective data from predominantly single-center cohorts; prospective multi-center validation remains essential. Third, incorporating additional potentially relevant factors such as detailed imaging biomarkers, genetic markers, or more detailed inflammatory profiling might improve predictive accuracy. Fourth, although inflammatory biomarker selection prioritized NLR based on existing evidence, future research is needed to directly compare a broader panel of indices (such as PLR, LMR, SII, PIV, and SIRI). Fifth, a complete-case analysis approach was used for handling missing data. Although comparative analysis demonstrated no systematic differences between patients excluded for missing data and the final cohort, mitigating immediate selection bias concerns, future prospective studies should employ advanced techniques such as multiple imputation to further enhance robustness. Our model demonstrates utility in facilitating identifying for AIS patients after IVT at elevated risk of unfavorable 90-day prognosis.

This research establishes a clinically feasible model using five routinely available predictors for post-IVT adverse outcomes in AIS. Logistic Regression demonstrated superior overall performance compared to seven other machine learning algorithms, achieving optimal balance between discrimination, calibration, and clinical utility. SHAP analysis quantified individual predictor contributions, confirming NLR as the most influential risk determinant. Our validated model provides a stratification tool to identify high-risk patients, enabling personalized interventions to mitigate poor 3-month outcomes.

## Data Availability

The original contributions presented in the study are included in the article/Supplementary material, further inquiries can be directed to the corresponding authors.
