# Supplementary material for: Explainable machine learning model for predicting the outcome of acute ischemic stroke after intravenous thrombolysis
Source: Front Neurol. 2025 Sep 26;16:1668816. doi: 10.3389/fneur.2025.1668816 (PMC12510848; doi:10.3389/fneur.2025.1668816)
Supplement: Supplementary file 1 [file Table_1.DOCX]

Supplementary Material

# Supplementary Data

# **Supplementary Table 1.** Comparison of patients excluded due to missing data with the included data.

| **Variables** | **Total**  **(n = 1100)** | **Excluded**  **(n = 162)** | **Included**  **(n = 938)** | ***P* value** |
| --- | --- | --- | --- | --- |
|  |  |  |  |  |
| **Demographics** | | | | |
| Age, median (IQR) | 68 (59, 76) | 68 (59, 74) | 68 (59, 77) | 0.190 |
| Gender, (male, %) | 726 (66.00) | 108 (66.67) | 618 (65.88) | 0.917 |
| BMI, median (IQR) | 24.82 (22.75, 27.06) | 25.11 (22.49, 26.78) | 24.80 (22.85, 27.06) | 0.787 |
| **Previous History** | | | | |
| Hypertension, n (%) | 709 (64.46) | 104 (64.20) | 605 (64.50) | 0.941 |
| DM, n (%) | 257 (23.36) | 39 (24.07) | 218 (23.24) | 0.896 |
| CHD, n (%) | 171 (15.55) | 22 (13.58) | 149 (15.88) | 0.529 |
| AF, n (%) | 113 (10.27) | 16 (9.88) | 97 (10.34) | 0.857 |
| Previous stroke, n (%) | 314 (28.55) | 39 (24.07) | 275 (29.32) | 0.204 |
| Anticoagulant therapy, n (%) | 156 (14.18) | 21 (12.96) | 135 (14.39) | 0.719 |
| Smoking, n (%) | 381 (34.64) | 53 (32.72) | 328 (34.97) | 0.578 |
| Drinking, n (%) | 184 (16.72) | 31 (19.13) | 153 (16.31) | 0.374 |
| **Baseline Parameters** | | | | |
| SBP, median (IQR) | 152 (138, 166) | 154 (139, 175) | 151 (138, 165) | 0.065 |
| DBP, median (IQR) | 86 (78, 95) | 87 (79, 96) | 86 (78, 94) | 0.113 |
| OTT, median (IQR) | 184 (130, 236) | 179 (121, 229) | 185 (130, 237) | 0.116 |
| ASPECTS, median (IQR) | 8 (7, 8) | 8 (7, 8) | 8 (7, 8) | 0.115 |
| Baseline NIHSS score, median (IQR) | 6 (4, 11) | 6 (5, 10) | 6 (4, 11) | 0.679 |
| TOAST classification |  |  |  | 0.108 |
| Large-artery atherosclerosis, n (%) | 739 (67.20) | 103 (63.60) | 636 (67.80) |  |
| Cardioembolic, n (%) | 126 (11.50) | 12 (7.41) | 114 (12.15) |  |
| Small-artery occlusion, n (%) | 224 (20.40) | 43 (26.50) | 181 (19.30) |  |
| Other etiology, n (%) | 10 (0.91) | 4 (2.47) | 6 (0.64) |  |
| Undetermined etiology, n (%) | 1 (0.09) | 0 (0.00) | 1 (0.11) |  |
| **Laboratory Data** | | | | |
| NLR, median (IQR) | 2.93 (1.92, 4.80) | 2.82 (1.85, 4.57) | 2.94 (1.95, 4.83) | 0.298 |
| Platelets, median (IQR) | 202 (164, 236) | 208 (170, 246) | 199 (162, 235) | 0.114 |
| Eosinophils, median (IQR) | 0.09 (0.04, 0.15) | 0.10 (0.04, 0.17) | 0.08 (0.04, 0.15) | 0.432 |
| Albumin, median (IQR) | 42.1 (39.2, 44.9) | 41.8 (38.7, 44.8) | 42.2 (39.2, 44.9) | 0.419 |
| Haemoglobin, median (IQR) | 141 (130, 152) | 141.00 (132, 152) | 141 (130, 152) | 0.811 |
| RDW, median (IQR) | 12.9 (12.4, 13.4) | 12.9 (12.5, 13.5) | 12.9 (12.4, 13.4) | 0.671 |
| TC, median (IQR) | 4.48 (3.91, 5.04) | 4.48 (4.04, 5.09) | 4.48 (3.91, 5.01) | 0.540 |
| TG, median (IQR) | 1.36 (0.98, 1.65) | 1.45 (1.08, 1.88) | 1.36 (0.97, 1.64) | 0.098 |
| HDL, median (IQR) | 1.07 (0.91, 1.17) | 1.05 (0.90, 1.14) | 1.07 (0.91, 1.17) | 0.349 |
| LDL, median (IQR) | 2.56 (2.12, 3.00) | 2.56 (2.12, 2.95) | 2.56 (2.12, 3.00) | 0.499 |
| CRP, median (IQR) | 1.50 (0.60, 4.50) | 1.50 (0.90, 4.90) | 1.55 (0.60, 4.50) | 0.582 |
| UA, median (IQR) | 305 (254, 365) | 296 (246, 359) | 308 (255, 366) | 0.487 |
| AST, median (IQR) | 24 (20, 29) | 25 (21, 29) | 24 (20, 29) | 0.465 |
| ALT, median (IQR) | 20 (15, 29) | 20 (16, 28) | 20 (15, 29) | 0.237 |
| GGT, median (IQR) | 22 (15, 35) | 21 (16, 33) | 23 (15, 35) | 0.350 |
| HbA1c, median (IQR) | 6.10 (5.60, 6.65) | 6.10 (5.60, 6.65) | 6.08 (5.60, 6.65) | 0.971 |
| Blood glucose, median (IQR) | 5.67 (4.95, 7.38) | 5.72 (5.17, 7.21) | 5.65 (4.90, 7.43) | 0.327 |

BMI, Body Mass Index; DM, diabetes mellitus; AF, Atrial fibrillation; CHD, coronary heart disease; OTT, Onset-to-treatment; NIHSS, National Institutes of Health Stroke Scale; SBP, systolic blood pressure; DBP, diastolic blood pressure; ASPECTS, Alberta Stroke Program Early CT Score; TOAST, Trial of ORG 10172 in Acute Stroke Treatment; IVT, intravenous thrombolysis; RDW, red cell distribution width; LDL, low-density lipoprotein; HDL, high-density lipoprotein;NLR, neutrophil-to-lymphocyte ratio.

# **Supplementary Table 2.** Comparison of baseline data between the two datasets.

| **Variables** | **Total (n = 938)** | **Training data**  **(n = 656)** | **Test data (n = 282)** | ***P* value** |
| --- | --- | --- | --- | --- |
|  |  |  |  |  |
| **Demographics** | | | | |
| Age, median (IQR) | 68 (59, 77) | 68 (59, 76) | 69(59, 77) | 0.460 |
| Gender, (male, %) | 618 (65.88) | 423 (64.48) | 195 (69.15) | 0.167 |
| BMI, median (IQR) | 24.80 (22.85, 27.06) | 24.99 (22.83, 27.34） | 24.61 (22.86, 26.71) | 0.214 |
| **Previous History** | | | | |
| Hypertension, n (%) | 605 (64.50) | 424 (64.63) | 181 (64.18) | 0.895 |
| DM, n (%) | 218 (23.24) | 153 (23.32) | 65 (23.05) | 0.928 |
| CHD, n (%) | 149 (15.88) | 98 (14.94) | 51 (18.09) | 0.227 |
| AF, n (%) | 97 (10.34) | 69 (10.52) | 28 (9.93) | 0.786 |
| Previous stroke, n (%) | 275 (29.32) | 203 (30.95) | 72 (25.53) | 0.095 |
| Anticoagulant therapy, n (%) | 135 (14.39) | 99 (15.09) | 36 (12.77) | 0.352 |
| Smoking, n (%) | 328 (34.97) | 230 (35.06) | 98 (34.75) | 0.927 |
| Drinking, n (%) | 153 (16.31) | 106 (16.16) | 47 (16.67) | 0.847 |
| **Baseline Parameters** | | | | |
| SBP, median (IQR) | 151 (138, 165) | 150 (137, 164) | 150(139, 164) | 0.759 |
| DBP, median (IQR) | 86 (78, 94) | 86 (78, 94) | 85(78, 94) | 0.850 |
| OTT, median (IQR) | 185 (130, 237) | 183(129, 236) | 190 (136, 238) | 0.266 |
| ASPECTS, median (IQR) | 8 (7, 8) | 7 (7, 8) | 8 (7, 8) | 0.268 |
| Baseline NIHSS score, median (IQR) | 6 (4, 11) | 6 (4, 11) | 6(4, 11) | 0.901 |
| TOAST classification |  |  |  | 0.960 |
| Large-artery atherosclerosis, n (%) | 636 (67.80) | 446 (67.99) | 190 (67.38) |  |
| Cardioembolic, n (%) | 114 (12.15) | 79 (12.04) | 35 (12.41) |  |
| Small-artery occlusion, n (%) | 181 (19.30) | 125 (19.05) | 56 (19.86) |  |
| Other etiology, n (%) | 6 (0.64) | 5 (0.76) | 1 (0.35) |  |
| Undetermined etiology, n (%) | 1 (0.11) | 1 (0.15) | 0 (0.00) |  |
| **Laboratory Data** | | | | |
| Neutrophil, median (IQR) | 4.88 (3.63, 6.47) | 4.87 (3.68, 6.55) | 4.88 (3.59, 6.36) | 0.899 |
| Lymphocyte, median (IQR) | 1.6 (1.2, 2.2) | 1.6 (1.2, 2.3) | 1.6 (1.2, 2.1) | 0.455 |
| NLR, median (IQR) | 2.94 (1.95, 4.83) | 2.92 (1.92, 4.90) | 2.99 (2.02, 4.80) | 0.658 |
| Platelets, median (IQR) | 199 (162, 235) | 198(162, 235) | 201(163, 233) | 0.741 |
| Eosinophils, median (IQR) | 0.08 (0.04, 0.15) | 0.08 (0.04, 0.15) | 0.09 (0.04, 0.14) | 0.727 |
| Albumin, median (IQR) | 42.2 (39.2, 44.9) | 42.2 (39.4, 44.9) | 42.1 (39.0, 44.9) | 0.727 |
| Haemoglobin, median (IQR) | 141 (130, 152) | 141 (129, 152) | 141 (132, 152) | 0.828 |
| RDW, median (IQR) | 12.9 (12.4, 13.4) | 12.9 (12.4, 13.4) | 12.9 (12.4, 13.4) | 0.339 |
| TC, median (IQR) | 4.48 (3.91, 5.01) | 4.45 (3.86, 4.99) | 4.48 (3.98, 5.05) | 0.489 |
| TG, median (IQR) | 1.36 (0.97, 1.64) | 1.36 (0.98, 1.64) | 1.36 (0.96, 1.62) | 0.842 |
| HDL, median (IQR) | 1.07 (0.91, 1.17) | 1.07 (0.91, 1.17) | 1.06 (0.91, 1.18) | 0.603 |
| LDL, median (IQR) | 2.56 (2.12, 3.00) | 2.56 (2.11, 3.00) | 2.56 (2.15, 3.00) | 0.897 |
| CRP, median (IQR) | 1.55 (0.60, 4.50) | 1.50 (0.60, 4.32) | 1.65 (0.50, 4.77) | 0.925 |
| UA, median (IQR) | 308 (255, 366) | 309 (256, 369) | 304(251, 360) | 0.236 |
| AST, median (IQR) | 24 (20, 29) | 24 (20, 30) | 24 (20, 29) | 0.964 |
| ALT, median (IQR) | 20 (15, 29) | 20 (15, 28) | 20 (15, 29) | 0.781 |
| GGT, median (IQR) | 23 (15, 35) | 23 (15, 35) | 23 (16, 36) | 0.491 |
| HbA1c, median (IQR) | 6.08 (5.60, 6.65) | 6.00 (5.60, 6.65) | 6.10 (5.60, 6.65) | 0.757 |
| Blood glucose, median (IQR) | 5.65 (4.90, 7.43) | 5.67 (4.92, 7.38) | 5.62 (4.89, 7.50) | 0.884 |

BMI, Body Mass Index; DM, diabetes mellitus; AF, Atrial fibrillation; CHD, coronary heart disease; OTT, Onset-to-treatment; NIHSS, National Institutes of Health Stroke Scale; SBP, systolic blood pressure; DBP, diastolic blood pressure; ASPECTS, Alberta Stroke Program Early CT Score; TOAST, Trial of ORG 10172 in Acute Stroke Treatment; IVT, intravenous thrombolysis; RDW, red cell distribution width; LDL, low-density lipoprotein; HDL, high-density lipoprotein;NLR, neutrophil-to-lymphocyte ratio.

**Supplementary Table 3.** The coefficients of the Lasso regression.

| **Variables** | **The minimum mean square error** | **The standard error of the minimum distance** |
| --- | --- | --- |
| (Intercept) | -1.77 | -1.419 |
| Atrial fibrillation | 0.502 | 0.227 |
| Anticoagulant therapy | 0.0 | 0.0 |
| TOAST classification | 0.0 | 0.0 |
| History of smoke | 0.0 | 0.0 |
| History of alcohol | 0.0 | 0.0 |
| NLR | 0.746 | 0.617 |
| Blood glucose | 0.117 | 0.014 |
| Admission NIHSS | 0.376 | 0.329 |
| ASPECTS | -0.243 | -0.173 |
| OTT | 0.01 | 0.0 |
| Age | 0.0 | 0.0 |
| SBP | 0.0 | 0.0 |
| Eosinophils | 0.0 | 0.0 |
| RDW | 0.0 | 0.0 |
| PLT | 0.0 | 0.0 |
| Albumin | 0.0 | 0.0 |
| HDL | 0.0 | 0.0 |

TOAST, Trial of ORG 10172 in Acute Stroke Treatment; NLR, neutrophil-to-lymphocyte ratio; NIHSS, National Institutes of Health Stroke Scale; ASPECTS, Alberta Stroke Program Early CT Score; OTT, Onset-to-treatment; SBP, systolic blood pressure; RDW, red cell distribution width; HDL, high-density lipoprotein.
